# Comparative Analysis of the Association of Biomarkers of Endothelial Dysfunction and Systemic Inflammation in Patients with Coronary Artery Disease with the Presence/Absence of Personality Type D

**DOI:** 10.3390/jcm15135227

**Published:** 2026-07-03

**Authors:** Alexey N. Sumin, Natalia N. Zagorskaya, Natalia A. Bezdenezhnykh, Anna V. Shcheglova, Yaroslav I. Bryukhanov, Anna V. Sinitskaya

**Affiliations:** 1 Department of Clinical Cardiology, Federal State Budgetary Institution “Research Institute for Complex Issues of Cardiovascular Disease”, 6, Blvd. Named Academician L.S. Barbarasha, Kemerovo 650002, Russia; n_zagorskaya@mail.ru (N.N.Z.); n_bez@mail.ru (N.A.B.); 2 Department of Innovative and Clinical Research, Federal State Budgetary Institution “Research Institute for Complex Issues of Cardiovascular Disease”, 6, Blvd. Named Academician L.S. Barbarasha, Kemerovo 650002, Russia; 3 Laboratory of Genomic Medicine, Department of Experimental Medicine, Federal State Budgetary Institution “Research Institute for Complex Issues of Cardiovascular Disease”, 6, Blvd. Named Academician L.S. Barbarasha, Kemerovo 650002, Russia

**Keywords:** type D personality, pathogenetic mechanisms, biomarker association, coronary heart disease

## Abstract

**Background**: The aim of this study was to comprehensively analyze the relationships within a multimodal biomarker panel, including endothelial function indicators, markers of systemic inflammation and myocardial stress, metabolic homeostasis parameters, and an indicator of microstructural damage to nerve tissue in CAD patients with or without type D personality. **Methods**: This exploratory, cross-sectional, observational study included 72 patients with coronary artery disease. All patients underwent psychological testing (evaluation of type D personality and determination of depression and anxiety levels) and biomarker measurements. The multimodal biomarker panel included measurements of metabolic homeostasis parameters (glucose, total cholesterol, creatinine, insulin, 1,5-anhydroglucitol), markers of systemic inflammation (CRP, IL-6), myocardial stress (NTproBNP), endothelial function parameters (eNOS, EDN1, ADMA, VEGF), and an indicator of microstructural damage to nerve tissue (S100B protein). **Results**: Biomarker levels revealed no statistically significant differences between the groups with and without personality type D. In personality type D, a direct correlation was found between the level of the brain tissue damage marker S100B and eNOS concentration (R = 0.578; *p* = 0.006), which was not observed in non-type D. In patients with personality type D, a significant inverse correlation was confirmed between ADMA and creatinine levels (R = −0.524; *p* = 0.015). In individuals with non-type D personality, a direct correlation was established between total cholesterol levels and VEGF (R = 0.342; *p* = 0.014). **Conclusions**: In patients with coronary heart disease, psychological distress (type D) is associated not with an isolated change in biomarker concentrations but with a transformation of the entire structure of their relationships. Personality type D is characterized by a transition from the physiological autonomy of systems to the formation of pathogenetic relationships between them, indicating a decrease in adaptive reserve.

## 1. Introduction

A patient-centered approach is a trend in modern medicine, which includes improving clinical practice through personalized approaches [1]. Psychological factors can determine not only the quality of life of patients with cardiovascular pathology [2] but also influence the clinical course of the disease [3]. Among such psychological factors, personality type D has recently been identified, characterized by a combination of excessive negative affectivity in response to stressors and a suppressed reaction to them in social interactions [4]. Individuals with this personality type are predisposed to developing psychological distress, manifested, among other things, by such a well-known psychosocial risk factor as depression [5]. Assessing and identifying individuals with personality type D allows for improving the psychosocial profile of patients before the onset of depression, that is, applying a personalized preventive approach. An additional feature of personality type D is its negative impact on both the quality of life of patients [6] and prognosis, particularly in patients with coronary artery disease (CAD) [7,8].

Although the negative impact of personality type D on prognosis has been proven, questions regarding the possible pathogenetic mechanisms of this impact remain unclear. In addition to behavioral factors [9], which include a negative attitude toward recommendations, poor adherence to therapy, and a tendency toward a less healthy lifestyle, inappropriate physiological responses to stress are also significant in such patients. It is hypothesized that the pathophysiological mechanism of the negative impact of personality type D on the prognosis of cardiovascular diseases is realized through chronic activation of the amygdala–hypothalamic–pituitary–adrenal axis [10]. Thus, it is known that CAD patients with type D exhibit elevated cortisol levels [11], an inadequate hemodynamic response to stress [12], and a tendency toward increased arterial wall stiffness under stress [13]. The influence of personality type D on markers of endothelial function in CAD patients is also known [14]. Nevertheless, it has not yet been possible to fully trace the mechanisms of the entire pathogenetic chain (from the neurohumoral response to stress to damage to the vascular bed and target organs).

Persistent psychological distress initiates a cascade of neurohumoral changes: hypercatecholaminemia and hypercortisolemia, and, as a result, resistance to central cortisol receptors, which acts as a trigger for immunocompetent cells. This results in a systemic increase in the concentration of proinflammatory cytokines (IL-1, IL-6, TNF-α, etc.) in the blood [15] and the initiation of oxidative stress processes in tissues [16]. When critical concentrations of proinflammatory agents are reached, they can compromise the integrity of the blood–brain barrier (BBB), triggering neuroinflammation and microstructural damage to cerebral tissue [17]. In this regard, the S100B marker serves as a sensitive indicator of CNS involvement in a systemic pathological process [18,19].

It is known that patients with depression exhibit elevated circulating ADMA levels, which contribute to a decrease in eNOS production. ADMA accumulation is believed to explain the increased risk of coronary heart disease in major depressive disorder [20]. It cannot be ruled out that in individuals with type D personality, a relationship may develop between the activity of neuroinflammation and the state of the microvascular bed (also susceptible to the damaging effects of high concentrations of proinflammatory cytokines); and, consequently, the interconnected functioning of the humoral regulation system of the endothelial response (eNOS, ADMA) with neuronal and glial damage. In this context, in individuals with personality type D, of interest are not only the dynamic changes in the vasodilating function of the endothelium, regulated, in particular, by eNOS and ADMA, but also the mechanisms of structural and functional reorganization of the vascular bed, regulated, among other things, by the powerful vasoconstrictor endothelin-1 (EDN1) and the vascular growth factor VEGF.

In parallel, persistent systemic expression of proinflammatory cytokines (IL-6 and other proinflammatory agents) and the associated increased synthesis of CRP form the background of chronic “sluggish” systemic inflammation, which acts as an additional factor in sympathetic activation [21] and myocardial damage [22,23]. In this context, assessing the level of NTproBNP is of interest. In the framework of our study, this peptide is considered not only as a marker of heart failure [24] but also as an indicator of myocardial damage arising from other causes [25], one of which may be chronic sympathetic hyperstimulation. We hypothesize that chronic catecholamine exposure in patients with type D personality may lead to myocardial functional overstrain and, possibly, microvascular ischemia. This, combined with systemic inflammation, links distress, systemic inflammation, and cardiovascular damage into a common pathogenetic chain.

We also assess glucose and cholesterol levels in the subjects, as we anticipate that changes in metabolic status may be an additional factor exacerbating the severity of diseases in individuals with type D personality. Stress-induced hypercortisolemia leads to changes in carbohydrate and lipid metabolism [26,27], manifesting as chronic hyperglycemia, hyperinsulinemia, and dyslipidemia (caused by the activation of lipolysis and increased synthesis of low-density lipoproteins (LDLs)). This metabolic imbalance accelerates atherogenesis and progresses to target organ damage. In this situation, assessing creatinine levels is also important: since systemic changes can lead to occult kidney damage, it is necessary to assess their functional integrity. Furthermore, the choice of creatinine as a key marker in our study was dictated by the need to analyze excretory processes as a mechanism for eliminating vasoactive metabolites (e.g., EDN1 and ADMA). This complements a comprehensive assessment of systemic homeostasis in this patient population.

Given the above, the aim of this study was to comprehensively analyze the relationships within a multimodal biomarker panel, including endothelial function indicators, markers of systemic inflammation and myocardial stress, metabolic homeostasis parameters, and an indicator of microstructural damage to nerve tissue in CAD patients with or without personality type D.

## 2. Methods

### 2.1. Study Design and Setting

This study was conducted as an exploratory, cross-sectional, observational study in CAD patients admitted for elective percutaneous intervention at the Research Institute for Complex Issues of Cardiovascular Diseases. Consecutive admissions between May and October 2025 were considered for inclusion. The study complies with the Declaration of Helsinki and is presented in accordance with the STROBE guidelines for cross-sectional studies. The study protocol was approved by the local ethics committee (Protocol No. 4, dated 10 March 2025).

### 2.2. Participants

Study inclusion criteria:

- Adult (over 18 years of age) male and female patients with no age limit.
- Verified coronary artery disease (CAD).
- Hospitalization in the department to determine the strategy for myocardial revascularization and elective percutaneous intervention (PCI).
- Written informed consent to participate in the study.

The exclusion criteria were formulated taking into account the need to minimize external pathogenetic effects on the profile of the studied biomarkers:

- Oncological and hematological diseases: Active oncological process (including within the last 5 years), as well as a history of courses of cytostatic chemotherapy or radiation therapy to the mediastinum (including lymphoproliferative diseases). These conditions and their treatment methods distort the levels of angiogenesis factors (VEGF), cause radiation-induced vascular fibrosis, and modify the endothelial secretion profile (EDN1, ADMA, eNOS).
- Systemic and autoimmune processes: Systemic connective tissue diseases (e.g., SLE) and active infectious diseases (including HIV infection) due to the presence of pronounced nonspecific systemic inflammation (IL-6, CRP).
- Immunosuppressive therapy: History of organ transplantation and concomitant therapy (cytostatics, systemic glucocorticoids) that can mask true endothelial dysfunction and inflammatory activity.
- Severe somatic pathology: Severe renal failure (to exclude false-positive results for creatinine and NTproBNP levels), as well as acute conditions at the time of inclusion (acute coronary syndrome, decompensated heart failure).

**Figure 1:**
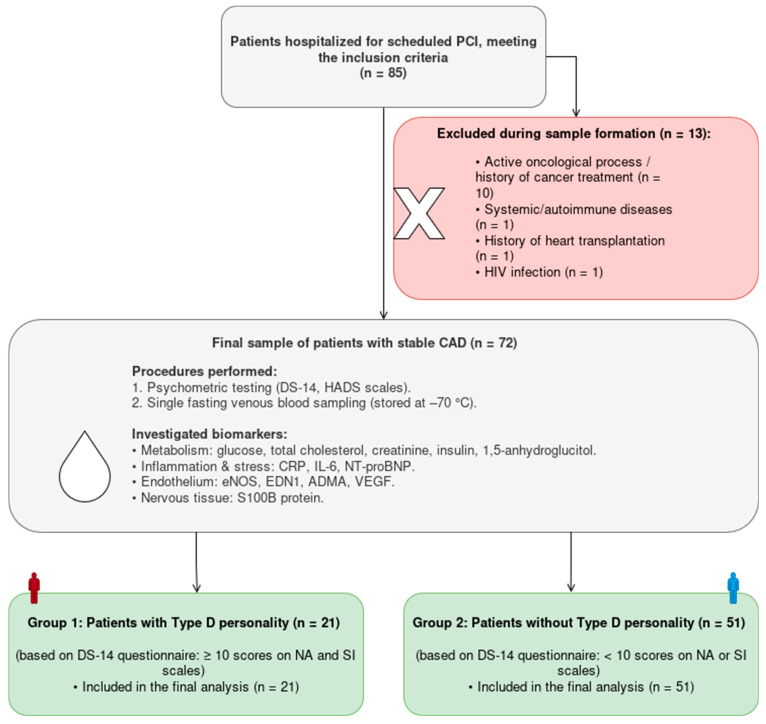
Flow diagram of the study participants.

The study initially included 85 patients admitted for elective PCI. During the final sample selection process for statistical analysis, 13 patients were excluded due to active oncological disease or a history of aggressive special treatment (*n* = 10), systemic diseases (*n* = 1), heart transplantation (*n* = 1), or HIV infection (*n* = 1). The final sample included in the analysis, which included a complete set of data on the studied biomarkers and psychological testing, consisted of 72 patients (Figure 1).

### 2.3. Psychometric Testing

To assess the patients’ psychological profile, standardized questionnaires validated for the Russian-speaking population were used:

#### 2.3.1. Determining Personality Type D (Distressed Personality)

The Russian-language version of the DS-14 questionnaire was used [28] (Cronbach’s α for the NA and SI scales was 0.78 and 0.74, respectively). The instrument includes 14 questions divided into two scales: negative affectivity (NA) and social inhibition (SI). Responses were assessed on a 5-point Likert scale (from 0 (not true) to 4 (absolutely true). The presence of Type D was determined by scoring 10 or more points on each scale. Based on the test results, patients were divided into a group with Type D personality (*n* = 20) and a group without it (*n* = 52).

#### 2.3.2. Assessment of Anxiety and Depression Levels

The Hospital Anxiety and Depression Scale (HADS) was used. This questionnaire contains 14 items comprising two subscales: “anxiety” (HADS-A) and “depression” (HADS-D). Each item was rated on a scale from 0 to 3 points depending on the severity of symptoms. The Russian version of the scale demonstrates high internal consistency (overall α = 0.90; α for anxiety = 0.86; α for depression = 0.84). The total score for each subscale was interpreted as an indicator of the severity of the respective disorders.

### 2.4. Collection of Biological Material

Venous blood samples for laboratory testing were collected during the patients’ inpatient stay (in the morning, on an empty stomach). Given the stable course of coronary artery disease, biopsy samples were collected once, either during preoperative preparation or in the early postoperative period after complete stabilization of hemodynamic and clinical parameters. Centrifuged samples were stored at −70 °C until laboratory analysis.

The multimodal biomarker panel included measurements of metabolic homeostasis parameters (glucose, total cholesterol, creatinine, insulin, 1,5-anhydroglucitol), markers of systemic inflammation (CRP, IL-6), myocardial stress (NTproBNP), endothelial function parameters (eNOS, EDN1, ADMA, VEGF), and an indicator of microstructural damage to nerve tissue (S100B protein).

### 2.5. Statistical Analysis

Results were processed using the Statistica 10.0 software package (StatSoft Inc., Tulsa, OK, USA). Given the non-normal distribution of most of the study variables (tested using the Shapiro–Wilk test), quantitative data are presented as median (Me) and interquartile range (25th and 75th percentiles).

For intergroup comparisons in independent samples, the Mann–Whitney U test was used. Analysis of relationships between quantitative parameters was performed using the Spearman rank correlation (R) method. To increase the statistical power of the study and minimize the risk of multiple comparison errors (Type I errors), the 13 laboratory markers studied were divided into three functional domains based on their pathogenetic role:

1. Domain of markers linking neurodestruction and endothelial dysfunction (S100B, eNOS, ADMA, EDN1, VEGF)—to assess the relationship between markers of nerve tissue damage and endothelial function and angiogenesis.
2. Domain of markers of systemic inflammation and myocardial dysfunction (CRP, IL-6, NTproBNP, VEGF)—to analyze the systemic inflammatory response and hemodynamic load on the myocardium. Given the role of VEGF as a mediator induced by inflammatory cytokines, this indicator was additionally included in the systemic inflammation domain analysis.
3. Metabolic status block (glucose, insulin, 1.5 AG, total cholesterol, creatinine)—to assess carbohydrate and lipid metabolism, and excretory function.

Within these blocks, false-positive results were minimized using the Benjamini–Hochberg false discovery rate (FDR) method. For statistically valid a priori hypotheses aimed at assessing key pathogenetic interactions (S100B-eNOS, ADMA-creatinine, and VEGF-cholesterol relationships), correction for multiple comparisons was not applied.

Results were considered statistically significant at *p* < 0.05 and *p_fdr_* < 0.05.

## 3. Results

### 3.1. Clinical and Demographic Characteristics of the Comparison Groups

The final sample consisted of 72 patients, of which 21 (29.17%) had verified personality type D, while 51 (70.83%) did not have type D. The groups were comparable in terms of key demographic parameters (Table 1): the median age in the group with personality type D was 67.0 years, compared to 68.0 years in the group without (*p* = 0.901). Gender composition also showed no statistically significant differences (the proportion of men in the type D group was 47.62%, compared to 60.78% in the group without; *p* = 0.305). Analysis of anthropometric data and comorbidities revealed no significant differences in body mass index (*p* = 0.965), diabetes mellitus and prediabetes (*p* = 0.459), history of myocardial infarction (*p* = 0.574), and acute cerebrovascular accident (*p* = 0.271). Detailed characteristics of the patient cohort are presented in Table 1.

The severity of patients’ cardiac symptoms, assessed by angina functional class (FC) and CHF stage, did not differ between the two samples (the median values for these parameters were identical). In the structure of heart failure, patients with preserved ejection fraction prevailed (85.71% in the group with personality type D and 74.51% in the comparison group D, *p* = 0.495).

However, significant differences in psychoemotional status were identified. In patients with personality type D, the level of depression on the HADS scale was significantly higher than in the comparison group (7.0 [6.0; 9.5] versus 5.0 [2.0; 7.0] points; *p* < 0.001). A similar, even more pronounced pattern was observed for the level of anxiety: 9.0 [7.0; 10.0] points in individuals with personality type D versus 5.0 [3.0; 6.0] points in the group without personality type D (*p* < 0.001). Clinically and subclinically expressed anxiety and depression were significantly more often recorded in the group with personality type D (for anxiety *p* < 0.001, for depression *p* = 0.043).

### 3.2. Comparison of Biomarker Levels in CAD Patients with and Without Personality Type D

A comparative analysis of biomarker levels based on the patients’ psychological profiles revealed no statistically significant differences between the groups for any of the studied parameters of the multimodal panel (Table 2). Levels of endothelial function, systemic inflammation, myocardial stress, and metabolic status were comparable in individuals with personality type D and in the comparison group (*p* > 0.05). Thus, the presence of psychological distress (type D) in the study sample was not associated with isolated quantitative changes in the concentrations of individual humoral factors.

### 3.3. Association for Biomarkers of Neurodestruction and Endothelial Dysfunction in CAD Patients with the Presence/Absence of Personality Type D

**Figure 2:**
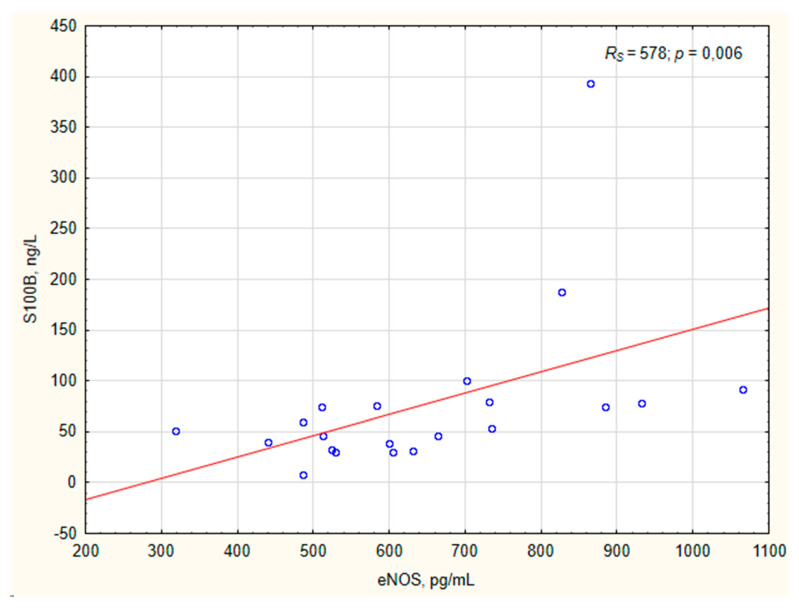
Correlation plot between S100B and eNOS in patients with type D personality.

When analyzing intrasystemic relationships within the neurovascular interaction domain (including S100B, eNOS, ADMA, EDN1, and VEGF) in the group of patients with personality type D, a direct moderate-strength correlation was found between the level of the brain tissue damage marker S100B and the concentration of eNOS (R = 0.578; *p* = 0.006) (Table 3, Figure 2).

Given the theoretical justification for the association between markers of neurodestruction and endothelial functional activity, this pair of indicators was considered the subject of an a priori formulated hypothesis, and therefore this relationship was recognized as statistically significant (R = 0.578; *p* = 0.006), despite the *p*_fdr_ value of 0.061 (Table 3). In contrast, in patients with non-D personality type, there were no correlations between S100B and endothelial function indices (in particular, for the S100B–eNOS pair R = 0.057) (Table 4). When analyzing the neurovascular interaction domain in the group of individuals with non-D personality type, no statistically significant correlations were found between the studied parameters (Table 4). Spearman’s correlation coefficients (R) between the microstructural damage to nerve tissue (S100B) and markers of endothelial function (eNOS, ADMA, EDN1), as well as vascular growth factor (VEGF), ranged from −0.205 to 0.169. This indicates the absence of an influence of neuroinflammation processes on the endothelial status and morphofunctional characteristics of the vascular bed as a whole in the group without personality type D.

### 3.4. Association for Biomarkers of Systemic Inflammation, Myocardial Dysfunction and Angiogenesis in CAD Patients with the Presence/Absence of Personality Type D

When analyzing the systemic inflammation and myocardial dysfunction domain in the group of patients with personality type D, no statistical relationships were found between the studied parameters. Levels of systemic inflammation markers (CRP, IL-6) and the myocardial functional stress index (NTproBNP), as well as vascular endothelial growth factor (VEGF), changed independently of each other. The absence of correlations within this block indicates that in the examined patients, the development of systemic inflammation and myocardial dysfunction, as well as the processes of angiogenesis, occur without the formation of unified pathogenetic mechanisms (Table 5).

**Figure 3:**
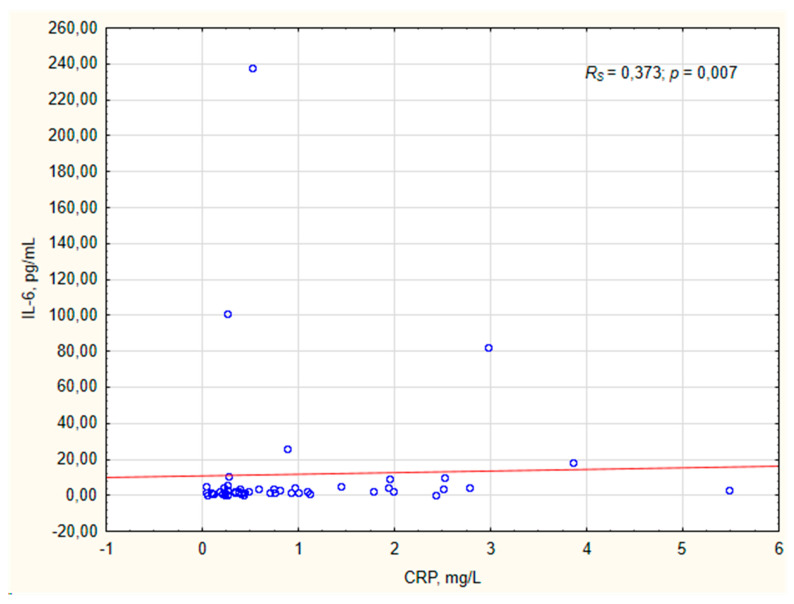
Correlation plot between IL-6 and CRP in patients with type not D personality.

**Figure 4:**
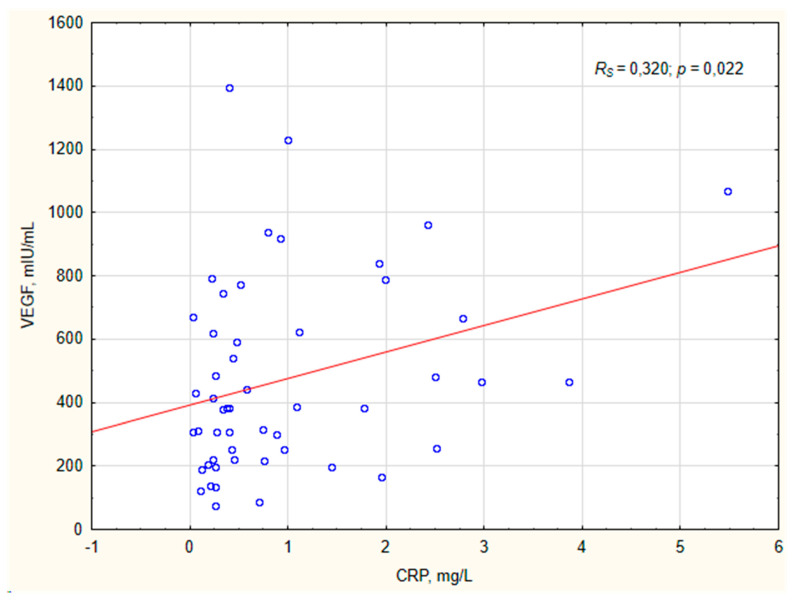
Correlation plot between VEGF and CRP in patients with type not D personality.

As for the analysis of the domain of markers of systemic inflammation, myocardial dysfunction and angiogenesis in individuals with non-D personality type, it revealed a different structure of relationships than in the group with type D personality (Table 6). In patients with an adaptive psychological profile (without type D), a moderate direct correlation was found between the levels of the main proinflammatory markers—CRP and IL-6 (R = 0.373; *p* = 0.007) (Figure 3). This relationship retained its statistical significance after introducing correction for multiple comparisons within the functional block (*p*_fdr_ = 0.043). In addition, a correlation was recorded between the level of CRP and the vascular growth factor VEGF (R = 0.320; *p* = 0.022) (Figure 4), which, after FDR correction, was still interpreted at the level of a statistically significant trend (*p*_fdr_ = 0.066). There were no relationships between these parameters and the NTproBNP level in this group.

### 3.5. Association for Biomarkers of Metabolic Status in CAD Patients with the Presence/Absence of Personality Type D

In the analysis of the metabolic profile domain (including glucose, cholesterol, creatinine, insulin, and 1,5-anhydroglucitol levels), no statistically significant relationships were found in the group of patients with type D personality (Table 7). The only moderate correlation at baseline was between glucose and 1,5-anhydroglucitol levels (R = 0.483; *p* = 0.027). However, after correction for multiple comparisons within a functional block (10 pairwise correlations), this relationship lost statistical significance (*p*_fdr_ = 0.267). The remaining metabolic parameters, including insulin, cholesterol, and creatinine levels, did not demonstrate significant associations with each other.

Analysis of the metabolic profile domain in individuals with non-D personality type (Table 8) demonstrated a lack of stable intrasystemic relationships. The only weak correlation at baseline was between glucose and creatinine (R = −0.294; *p* = 0.036). However, after correction for multiple comparisons within a block (FDR), this association lost statistical significance (*p*_fdr_ = 0.360). The remaining metabolic status parameters, including cholesterol, insulin, and 1,5-anhydroglucitol levels, did not demonstrate significant associations. Thus, regardless of the psychological profile (type D or non-D), the studied metabolic status parameters are characterized by the absence of noticeable pathogenetic relationships.

### 3.6. Analysis of Target Pathogenetic Interactions (Testing of a Priori Hypotheses)

Taking into account the theoretical assumptions formulated during the study planning stage, statistical testing of three a priori hypotheses was conducted. Due to the targeted nature of the pairwise interaction data analysis, no correction for multiple comparisons was applied.

**Figure 5:**
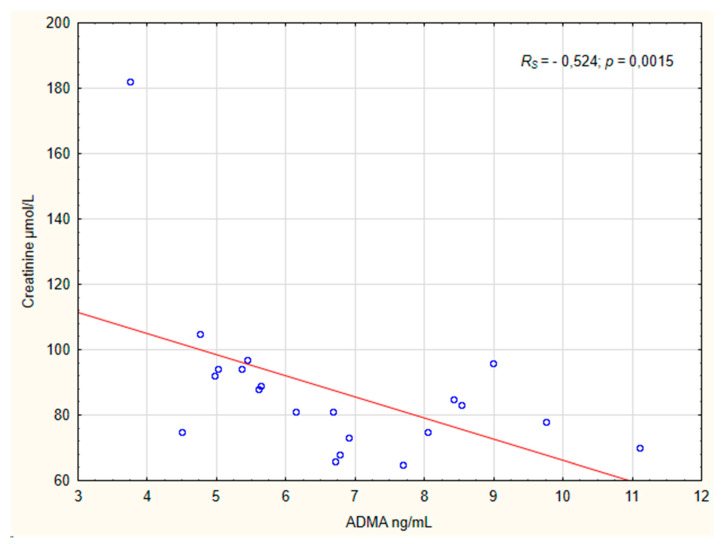
Correlation plot between creatinine and ADMA in patients with type D personality.

**Figure 6:**
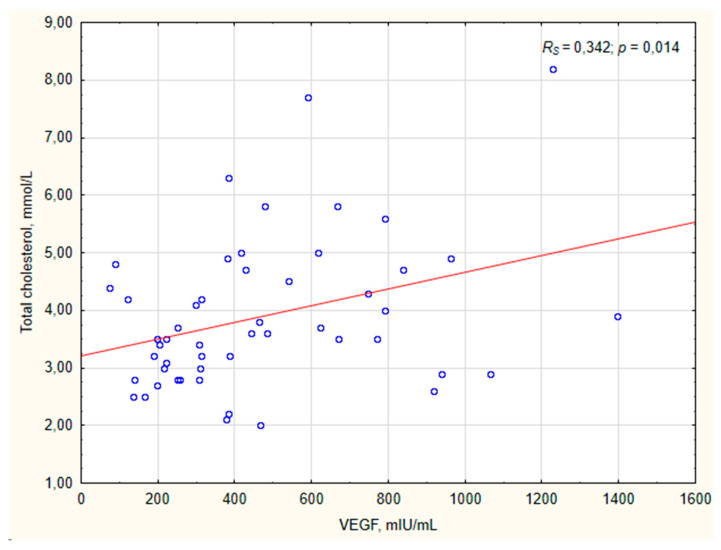
Correlation plot between total cholesterol and VEGF in patients with type not D personality.

1. Neurovascular interaction: An assessment of the relationship between the nerve tissue damage marker and endothelial functional activity revealed a direct moderate correlation between S100B and eNOS levels exclusively in the group of individuals with personality type D (R = 0.578, *p* = 0.006) (Figure 2). A similar relationship was absent in the comparison group (non-D personality type) (R = 0.057; *p* = 0.693).
2. Renal ADMA clearance: The second hypothesis concerned the relationship between the level of the endogenous NO synthase inhibitor and renal excretory function. In the group of patients with personality type D, a significant inverse correlation between ADMA and creatinine levels was confirmed (R = −0.524; *p* = 0.015) (Figure 5). In individuals with an adaptive psychological profile (non-D), no association between these indicators was recorded (R = −0.061; *p* = 0.669).
3. Adaptive angiogenesis: The third hypothesis was to assess the intensity of vascular formation in response to the activation of atherogenic mechanisms. In individuals with personality type non-D, a direct correlation was established between the level of total cholesterol and the VEGF factor (R = 0.342; *p* = 0.014) (Figure 6). In personality type D, a similar relationship between lipid levels and the angiogenesis marker was completely absent (R = 0.035; *p* = 0.880).

## 4. Discussion

The results obtained confirm the hypothesis of overstrain, with a probable tendency to subsequent decompensation, of adaptive mechanisms in individuals with personality type D [29]. The identified neurovascular interaction (S100B–eNOS), in our opinion, is of a compensatory nature: in response to microstructural damage to brain tissue, the production of nitric oxide through eNOS is activated [30] (which helps to strengthen microcirculation), as a protective factor and important link promoting reparation [31,32]. However, the presence of such a connection indicates that the endothelial system in this category of patients operates under severe functional stress, the chronic maintenance of which triggers a transition from adaptive endothelial activation to its persistent dysfunction [33]. In this case, this is determined by the degree of neuronal damage.

To date, it has been shown that personality type D is not only associated with the presence of sympathetic activation [10], but also that indicators SDNN and SDANN (key heart rate variability indices) mediate the influence of personality type D on the prognosis of patients with coronary artery disease after PCI [10]. The presence of central mechanisms of influence of type D personality is confirmed by the data of Meral HB et al. [34], who showed that among patients with fibromyalgia with signs of type D, higher central sensitization was observed.

In patients with coronary artery disease with personality type D, more pronounced endothelial dysfunction was revealed (flow-mediated dilation < 5.5%) than in patients without type D, both at baseline and during prospective follow-up for 12 months. At the same time, in type D, the decrease in the level of circulating CD34+/KDR+/CD45+dim endothelial progenitor cells was less significant (*p* = 0.07). Also, in patients with arterial hypertension, the presence of personality type D was associated with more pronounced changes in diastolic blood pressure to stress and vasodilator response during tests [35]. In addition, adolescents with type D personality showed a decrease in vascular elasticity [36].

In addition to studying the association of individual biomarkers with the presence of type D personality, complex associations are also being actively studied. A study by von Känel R et al. [21] showed that burnout and synergistic Type D personality traits were associated with changes in the level of reduction in pericoronal adipose tissue, consistent with an indirect effect through increased sympathetic activity. Lower levels of pericoronal adipose tissue reduction enhanced sympathetic associations with systemic inflammation, consistent with an outside-in mechanism. Lipid-rich perivascular adipose tissue may indicate an early stage of coronary artery vulnerability, leading the authors to suggest that psychosocial stress is associated with coronary artery biology before overt vascular inflammation [21].

Of interest is the relationship we found between the ADMA marker and creatinine levels. [37,38]. We believe that under conditions of systemic oxidative stress associated with chronic psychological distress [39,40], the activity of the DDAH (dimethylarginine dimethylaminohydrolase) enzyme, responsible for the metabolic degradation of ADMA, is suppressed [41]. Considering that the main expression of DDAH is concentrated in the tissues of the kidneys and liver [42], a decrease in its enzymatic potential increases the dependence of the elimination of this vasoconstrictor on the excretory function of the kidneys [43] (which is confirmed by the identified relationship between ADMA and creatinine).

Thus, a multidirectional load is exerted on the endothelium. On the one hand, there is an adaptive attempt at vasodilation (via the S100B–eNOS relationship we identified). Although the literature considers S100B to be an inducer of vascular damage and endothelial dysfunction [44,45], our data suggest its temporary compensatory role. On the other hand, this adaptation can be blocked by the action of a potent endogenous ADMA inhibitor, whose normal elimination mechanisms are disrupted. If, in individuals with an adaptive profile (non-D type), the vascular mechanisms retain sufficient lability, then in personality type D, the situation is different: there is a pronounced relationship between endothelial function and the processes of neuroinflammation and neurodegeneration [14,46,47]. In this case, in parallel with the accumulation of endogenous inhibitors, depletion of vasodilation resources develops, which deprives the vascular bed of compensatory flexibility [48]. All of the above can create conditions for the formation of persistent vasoconstriction in carriers of personality type D [8,49].

It has been previously shown that patients with coronary artery disease with type D had increased levels of TNF-α (*p* = 0.007), IL-6 (*p* = 0.049), total inflammation indices (*p* = 0.002), kynurenine (*p* = 0.008) and kynurenine/tryptophan ratio (*p* = 0.005) compared to a group of individuals without personality type D. Sequential multiple mediation analysis showed that type D personality may influence the formation of vulnerable plaques and subsequent serious cardiovascular events through inflammatory biomarkers [50]. It is known that under conditions of psychological distress, VEGF can play an important neuro- and vasculoprotective role, acting as an adaptive compensatory factor [51]. The absence of adaptive relationships between VEGF and markers of inflammation (CRP) and lipid metabolism (cholesterol) in individuals with personality type D, in contrast to the control group [52], indicates the rigidity of the angiogenic response. Under conditions of chronic neuroinflammation (high S100B) and endothelial stress (high ADMA), the “repair” potential of VEGF is insufficient [53]. Also noteworthy is the inadequate hemodynamic responses to stress [12] and reduced cortisol response during anticipation of psychosocial stress [54] in patients with coronary heart disease and personality type D.

It is also noteworthy that in the non-D personality type group, the correlation between glucose and 1,5-anhydroglucitol levels was completely absent, which was observed in individuals with type D in the primary analysis (before adjusting for multiple comparisons). It is possible that in individuals with an adaptive personality profile, glycemic fluctuations are shorter and less pronounced, and, therefore, the threshold at which a significant change in 1,5-anhydroglucitol concentration occurs is not reached [55]. In patients with type D, the metabolic system exhibits a tendency to form correlations, which may indicate a decrease in compensatory reserves of carbohydrate metabolism under conditions of psychological distress [56,57].

The observed functional independence of metabolic parameters in the control group, coupled with the preservation of flexible adaptive relationships (such as cholesterol-VEGF and CRP-VEGF), confirms the high potential for physiological resilience in individuals without type D [58,59]. At the same time, the tendency to develop pathogenetic dependencies even within the metabolic system, identified in type D carriers, emphasizes the systemic nature of homeostasis restructuring during psychological distress, which certainly requires further study in larger samples [60,61].

It should be noted that the group of patients with type D personality did not differ from the group without this type in most of the parameters studied when directly comparing the groups, contrary to some previous publications. In our opinion, this is due to the fact that type D is not a disease but rather a latent overstrain of regulatory systems. Under resting conditions (baseline), marker concentrations may remain stable and equal to those in the control group. However, the architecture of correlations is already restructured, as the functional relationships between the systems change.

The limitations of the study should be considered when evaluating its results. First, this study was conducted in only one center, which does not allow for its results to be extended to other centers and regions. Second, the study included a relatively small number of participants; however, this did not prevent statistically significant associations from being obtained. Third, this study used a dichotomous construct of Type D personality, which has been questioned in some publications (which propose assessing this type as a combination of continuous values of two subscales—negative affectivity and social inhibition) [62]. However, most recent publications predominantly use a dichotomous approach to the concept of Type D personality, which has confirmed, among other things, its independent predictive value [7,8,63]. Therefore, we considered it appropriate to use this approach in our analysis. It should also be understood that the design of our cross-sectional study does not allow us to make firm cause-and-effect statements. Furthermore, this study did not measure hormone levels. So, we cannot exclude the possibility that hormonal changes in people with type D affect the severity of the findings.

## 5. Conclusions

This study identified a specific systemic restructuring in patients with coronary heart disease and personality type D. It was found that psychological distress (type D) is associated not with isolated changes in biomarker concentrations but with a transformation of the entire structure of their relationships. Personality type D is characterized by a transition from the physiological autonomy of systems to the formation of pathogenetic relationships between them, indicating a decrease in adaptive reserve. A relationship between the brain damage marker S100B and endothelial nitric oxide synthase (eNOS) was also found exclusively in individuals with type D, suggesting a possible “spike” in the functioning of the nervous and vascular systems. This apparently makes the brain of these patients extremely vulnerable: the slightest damage to nervous tissue in them is transformed into stress in the regulation of endothelial function and the vascular bed.

Metabolic vulnerability of the endothelium is demonstrated by the observed dependence of ADMA levels on renal function (creatinine) only in type D, supporting the hypothesis of probable decompensation of enzymatic pathways that protect blood vessels. This makes endothelial homeostasis in these patients dependent on renal excretory function, creating conditions for the development of a “vicious cycle” of cardiorenal complications. Furthermore, individuals with non-D personality type retain protective intersystemic connections (VEGF with cholesterol and CRP), which ensure compensatory vascular growth in response to stress (and vasoconstriction), inflammation, and atherogenesis. The absence of these connections in type D personality, combined with a rigid metabolic profile, may indicate a deficiency of protective mechanisms and an increased risk of coronary heart disease progression, i.e., a deficiency of adaptive angiogenesis. However, the above considerations require confirmation in larger studies to clarify the pathogenetic associations.

## Figures and Tables

**Table 1:** General characteristics of the cohort of subjects depending on the presence/absence of personality type D.

Parameters (Me [LQ; UQ]) or *n* (%)	Type Not D (*n* = 51)	Type D (*n* = 21)	*p*
Age, years	68.0 [60.0; 75.0]	67.0 [66.0; 73.0]	0.901
Men	31 (60.78%)	10 (47.62%)	0.305
Disability	10 (19.61%)	6 (28.57%)	0.406
Smoking (current or history) (*n* = 65)	16 (34.04%)	9 (50.0%)	0.237
Angina pectoris FC (*n* = 69)	2.0 [1.0; 2.0]	2.0 [2.0; 2.0]	0.070
Angina pectoris FC (*n* = 69):			0.106
0	12 (24.49%)	1 (5.00%)	
I	4 (8.0%)	0 (0%)	
II	32 (65.31%)	18 (90.00%)	
III	1 (2.04%)	1 (5.00%)	
CHF stage	1.0 [1.0; 1.0]	1.0 [1.0; 1.0]	0.911
CHF stage:			0.816
0 (pre-heart failure stage)	4 (7.84%)	2 (9.52%)	
1	47 (92.16%)	19 (90.48%)	
CHF FC (*n* = 61)	2.0 [2.0; 2.0]	2.0 [2.0; 2.0]	0.995
CHF, FC (*n* = 61):			0.470
0	4 (8.00%)	2 (9.52%)	
1	2 (4.00%)	1 (4.76%)	
2	44 (88.00%)	17 (80.95%)	
3	0 (0%)	1 (4.76%)	
CHF by EF:			0.495
pre-heart failure stage	4 (7.84%)	2 (9.52%)	
HFpEF	38 (74.51%)	18 (85.71%)	
HFmrEF	5 (9.80%)	1 (4.76%)	
HFrEF	4 (8.0%)	0 (0%)	
History of MI	23 (45.10%)	11 (52.38%)	0.574
History of stroke	7 (13.73%)	1 (4.76%)	0.271
Body mass index, kg/m^2^	30.36 [27.41; 34.19]	30.74 [25.00; 33.66]	0.965
Carbohydrate metabolism disorders:			0.459
absent	29 (56.86%)	15 (71.43%)	
prediabetes	1 (1.96%)	0 (0%)	
DM	21 (41.18%)	6 (28.57%)	
HADS Depression subscale score (*n* = 71)	5.0 [2.0; 7.0]	7.0 [6.0; 9.5]	<0.001
HADS Anxiety subscale score	5.0 [3.0; 6.0]	9.0 [7.0; 10.0]	<0.001
Presence of depression (subclinical, clinically significant) by HADS (*n* = 71)	7 (13.73%)	7 (33.33%)	0.043
Presence of anxiety (subclinical, clinically significant) by HADS	8 (15.69%)	14 (66.67%)	<0.001
Beta-blockers	28 (54.9%)	11 (52.3%)	0.845
Calcium channel blockers	19 (37.3%)	6 (28.6%)	0.482
Statins	30 (58.8%)	12 (57.1%)	0.895
Angiotensin II receptor antagonists	13 (25.5%)	8 (38.1%)	0.285
Angiotensin-converting enzyme inhibitors	34 (66.7%)	13 (61.9%)	0.702
Aspirin	42 (82.4%)	16 (76.2%)	0.548
Clopidogrel	12 (23,5%)	5 (23.8%)	0.979

Note: Me [LQ; UQ]—median with lower and upper quartiles, FC—functional class, CHF—chronic heart failure, HFpEF—chronic heart failure with preserved ejection fraction (≥50%), HFmrEF—chronic heart failure with mildly reduced ejection fraction (41–49%), HFrEF—chronic heart failure with reduced ejection fraction (≤40%), MI—myocardial infarction, Stroke—acute cerebrovascular accident.

**Table 2:** Laboratory parameters of subjects depending on the presence/absence of personality type D.

Parameters (Me [LQ; UQ])	Type D (*n* = 21)	Type Not D (*n* = 51)	*p*
Glucose (mmol/L)	5.70 [5.20; 6.60]	5.30 [5.00; 6.40]	0.113
Total cholesterol (mmol/L)	3.70 [3.30; 4.40]	3.60 [2.90; 4.70]	0.719
Creatinine (µmol/L)	83.00 [75.00; 94.00]	86.00 [74.00; 105.00]	0.417
Insulin (µIU/mL)	7.40 [3.95; 23.07]	7.56 [3,68; 13.01]	0.476
CRP (mg/L)	0.52 [0.36; 1.75]	0.45 [0.25; 1.11]	0.389
IL-6 (pg/mL)	2.19 [1.50; 3.51]	2.24 [1.50; 4.31]	0.892
VEGF (mIU/mL)	386.30 [226.10; 690.80]	384.30 [222.70; 665.80]	0.761
NT-proBNP (pg/mL)	35.98 [28.33; 48.12]	42.28 [28.78; 71.06]	0.665
eNOS (pg/mL)	605.30 [512.60; 734.20]	583.80 [491.40; 761.80]	0.706
EDN1 (pg/mL)	29.61 [22,94; 34.00]	29.31 [25.29; 34.79]	0.407
ADMA (ng/mL)	6.68 [5.36; 8.04]	6.84 [5.54; 8.18]	0.439
1,5 AG (ng/mL)	540.20 [169.80; 699.20]	380.10 [141.60; 771.60]	0.809
S100B (ng/L)	53.98 [39.38; 78.42]	56.70 [41.08; 75.37]	0.901

Note: Me [LQ; UQ]—median with lower and upper quartiles, CRP—C-reactive protein, IL-6—interleukin 6, VEGF—vascular endothelial growth factor, NT-proBNP—N-terminal pro-B-type natriuretic peptide, eNOS—endothelial nitric oxide synthase, EDN1—endothelin-1, ADMA—asymmetric dimethylarginine, 1,5-AG—1,5-anhydroglucitol, S100B—S100 calcium-binding protein B.

**Table 3:** Spearman correlation analysis for markers of the relationship between neurodestruction and endothelial dysfunction in individuals with personality type D.

Parameter	VEGF (mIU/mL)	eNOS (pg/mL)	EDN1 (pg/mL)	ADMA (ng/mL)	S100B (ng/L)
VEGF (mIU/mL)	1.000	*R* = 0.217	*R* = 0.035	*R* = 0.296	*R* = 0.105
		*p* = 0.345	*p* = 0.880	*p* = 0.192	*p* = 0.650
		*p*_fdr_ = 0.575	*p*_fdr_ = 0.880	*p*_fdr_ = 0.562	*p*_fdr_ = 0.880
eNOS (pg/mL)	*R* = 0.217	1.000	*R* = 0.247	*R* = 0.060	*R* = 0.578
	*p* = 0.345		*p* = 0.281	*p* = 0.597	*p* = 0.006
	*p*_fdr_ = 0.575		*p*_fdr_ = 0.562	*p*_fdr_ = 0.880	*p*_fdr_ = 0.061
EDN1 (pg/mL)	*R* = 0.035	*R* = 0.247	1.000	*R* = 0.297	*R* = 0.259
	*p* = 0.880	*p* = 0.281		*p* = 0.190	*p* = 0.258
	*p*_fdr_ = 0.880	*p*_fdr_ = 0.562		*p*_fdr_ = 0.562	*p*_fdr_ = 0.562
ADMA (ng/mL)	*R* = 0.296	*R* = 0.060	*R* = 0.297	1.000	*R* = 0.050
	*p* = 0.192	*p* = 0.597	*p* = 0.190		*p* = 0.830
	*p*_fdr_ = 0.562	*p*_fdr_ = 0.880	*p*_fdr_ = 0.562		*p*_fdr_ = 0.880
S100B (ng/L)	*R* = 0.105	*R* = 0.578	*R* = 0.259	*R* = 0.050	1.000
	*p* = 0.650	*p* = 0.006	*p* = 0.258	*p* = 0.830	
	*p*_fdr_ = 0.880	*p*_fdr_ = 0.061	*p*_fdr_ = 0.562	*p*_fdr_ = 0.880	

Note: VEGF—vascular endothelial growth factor, eNOS—endothelial nitric oxide synthase, EDN1—endothelin-1, ADMA—asymmetric dimethylarginine, S100B—S100 calcium-binding protein B, *R*—Spearman’s rank correlation coefficient, *p*_fdr_—*p*-value adjusted for multiple comparisons using the Benjamini–Hochberg false discovery rate procedure.

**Table 4:** Spearman correlation analysis for markers of the relationship between neurodestruction and endothelial dysfunction in individuals with personality type not D.

Parameter	VEGF (mIU/mL)	eNOS (pg/mL)	EDN1 (pg/mL)	ADMA (ng/mL)	S100B (ng/L)
VEGF (mIU/mL)	1.000	*R* = 0.073	*R* = −0.144	*R* = 0.169	*R* = 0.014
		*p* = 0.609	*p* = 0.314	*p* = 0.236	*p* = 0.920
		*p*_fdr_ = 0.951	*p*_fdr_ = 0.951	*p*_fdr_ = 0.951	*p*_fdr_ = 0.951
eNOS (pg/mL)	*R* = 0.073	1.000	*R* = −0.205	*R* = −0.149	*R* = 0.057
	*p* = 0.609		*p* = 0.149	*p* = 0.296	*p* = 0.693
	*p*_fdr_ = 0.951		*p*_fdr_ = 0.951	*p*_fdr_ = 0.951	*p*_fdr_ = 0.951
EDN1 (pg/mL)	*R* = −0.144	*R* = −0.205	1.000	*R* = −0.009	*R* = −0.073
	*p* = 0.314	*p* = 0.149		*p* = 0.951	*p* = 0.613
	*p*_fdr_ = 0.951	*p*_fdr_ = 0.951		*p*_fdr_ = 0.951	*p*_fdr_ = 0.951
ADMA (ng/mL)	*R* = 0.169	*R* = −0.149	*R* = −0.009	1.000	*R* = −0.121
	*p* = 0.236	*p* = 0.296	*p* = 0.951		*p* = 0.397
	*p*_fdr_ = 0.951	*p*_fdr_ = 0.951	*p*_fdr_ = 0.951		*p*_fdr_ = 0.951
S100B (ng/L)	*R* = 0.014	*R* = 0.057	*R* = −0.073	*R* = −0.121	1.000
	*p* = 0.920	*p* = 0.693	*p* = 0.613	*p* = 0.397	
	*p*_fdr_ = 0.951	*p*_fdr_ = 0.951	*p*_fdr_ = 0.951	*p*_fdr_ = 0.951	

Note: VEGF—vascular endothelial growth factor, eNOS—endothelial nitric oxide synthase, EDN1—endothelin-1, ADMA—asymmetric dimethylarginine, S100B—S100 calcium-binding protein B, *R*—Spearman’s rank correlation coefficient, *p*_fdr_—*p*-value adjusted for multiple comparisons using the Benjamini–Hochberg false discovery rate procedure.

**Table 5:** Spearman correlation analysis for markers of systemic inflammation, myocardial dysfunction and angiogenesis in individuals with personality type D.

Parameter	CRP (mg/L)	IL-6 (pg/mL)	VEGF (mIU/mL)	NT-proBNP (pg/mL)
CRP (mg/L)	1.000	*R* = 0.045	*R* = −0.148	*R* = 0.091
		*p* = 0.845	*p* = 0.522	*p* = 0.695
		*p*_fdr_ = 0.978	*p*_fdr_ = 0.978	*p*_fdr_ = 0.978
IL-6 (pg/mL)	*R* = 0.045	1.000	*R* = −0.060	*R* = 0.156
	*p* = 0.845		*p* = 0.797	*p* = 0.500
	*p*_fdr_ = 0.978		*p*_fdr_ = 0.978	*p*_fdr_ = 0.978
VEGF (mIU/mL)	*R* = −0.148	*R* = −0.060	1.000	*R* = 0.006
	*p* = 0.522	*p* = 0.797		*p* = 0.978
	*p*_fdr_ = 0.978	*p*_fdr_ = 0.978		*p*_fdr_ = 0.978
NT-proBNP (pg/mL)	*R* = 0.091	*R* = 0.156	*R* = 0.006	1.000
	*p* = 0.695	*p* = 0.500	*p* = 0.978	
	*p*_fdr_ = 0.978	*p*_fdr_ = 0.978	*p*_fdr_ = 0.978	

Note: CRP—C-reactive protein, IL-6—interleukin 6, VEGF—vascular endothelial growth factor, NT-proBNP—N-terminal pro-B-type natriuretic peptide, *R*—Spearman’s rank correlation coefficient, *p*_fdr_—*p*-value adjusted for multiple comparisons using the Benjamini–Hochberg false discovery rate procedure.

**Table 6:** Spearman correlation analysis for markers of systemic inflammation, myocardial dysfunction and angiogenesis in individuals with personality type not D.

Parameter	CRP (mg/L)	IL-6 (pg/mL)	VEGF (mIU/mL)	NT-proBNP (pg/mL)
CRP (mg/L)	1.000	*R* = 0.373	*R* = 0.320	*R* = 0.034
		*p* = 0.007	*p* = 0.022	*p* = 0.813
		*p*_fdr_ = 0.043	*p*_fdr_ = 0.066	*p*_fdr_ = 0.848
IL-6 (pg/mL)	*R* = 0.373	1.000	*R* = 0.028	*R* = 0.147
	*p* = 0.007		*p* = 0.848	*p* = 0.304
	*p*_fdr_ = 0.043		*p*_fdr_ = 0.848	*p*_fdr_ = 0.608
VEGF (mIU/mL)	*R* = 0.320	*R* = 0.028	1.000	*R* = 0.028
	*p* = 0.022	*p* = 0.848		*p* = 0.844
	*p*_fdr_ = 0.066	*p*_fdr_ = 0.848		*p*_fdr_ = 0.848
NT-proBNP (pg/mL)	*R* = 0.034	*R* = 0.147	*R* = 0.028	1.000
	*p* = 0.813	*p* = 0.304	*p* = 0.844	
	*p*_fdr_ = 0.848	*p*_fdr_ = 0.608	*p*_fdr_ = 0.848	

Note: CRP—C-reactive protein, IL-6—interleukin 6, VEGF—vascular endothelial growth factor, NT-proBNP—N-terminal pro-B-type natriuretic peptide, *R*—Spearman’s rank correlation coefficient, *p*_fdr_—*p*-value adjusted for multiple comparisons using the Benjamini–Hochberg false discovery rate procedure.

**Table 7:** Spearman correlation analysis of metabolic status markers in individuals with personality type D.

Parameter	Glucose (mmol/L)	Total Cholesterol (mmol/L)	Creatinine (µmol/L)	Insulin (µIU/mL)	1,5 AG (ng/mL)
Glucose (mmol/L)	1.000	*R* = 0.132	*R* = −0.319	*R* = 0.332	*R* = 0.483
		*p* = 0.570	*p* = 0.158	*p* = 0.142	*p* = 0.027
		*p*_fdr_ = 0.814	*p*_fdr_ = 0.708	*p*_fdr_ = 0.708	*p*_fdr_ = 0.267
Total cholesterol (mmol/L)	*R* = 0.132	1.000	*R* = 0.056	*R* = 0.194	*R* = −0.026
	*p* = 0.570		*p* = 0.808	*p* = 0.400	*p* = 0.910
	*p*_fdr_ = 0.814		*p*_fdr_ = 0.910	*p*_fdr_ = 0.814	*p*_fdr_ = 0.910
Creatinine (µmol/L)	*R* = −0.319	*R* = 0.056	1.000	*R* = 0.295	*R* = −0.178
	*p* = 0.158	*p* = 0.808		*p* = 0.194	*p* = 0.441
	*p*_fdr_ = 0.708	*p*_fdr_ = 0.910		*p*_fdr_ = 0.708	*p*_fdr_ = 0.814
Insulin (µIU/mL)	*R* = 0.332	*R* = 0.194	*R* = 0.295	1.000	*R* = −0.055
	*p* = 0.142	*p* = 0.400	*p* = 0.194		*p* = 0.814
	*p*_fdr_ = 0.708	*p*_fdr_ = 0.814	*p*_fdr_ = 0.708		*p*_fdr_ = 0.910
1,5 AG (ng/mL)	*R* = 0.483	*R* = −0.026	*R* = −0.178	*R* = −0.055	1.000
	*p* = 0.027	*p* = 0.910	*p* = 0.441	*p* = 0.814	
	*p*_fdr_ = 0.267	*p*_fdr_ = 0.910	*p*_fdr_ = 0.814	*p*_fdr_ = 0.910	

Note: 1,5-AG—1,5-anhydroglucitol, *R*—Spearman’s rank correlation coefficient, *p*_fdr_—*p*-value adjusted for multiple comparisons using the Benjamini–Hochberg false discovery rate procedure.

**Table 8:** Spearman correlation analysis of metabolic status markers in individuals with personality type not D.

Parameter	Glucose (mmol/L)	Total Cholesterol (mmol/L)	Creatinine (µmol/L)	Insulin (µIU/mL)	1,5 AG (ng/mL)
Glucose (mmol/L)	1.000	*R* = −0.161	*R* = −0.294	*R* = 0.022	*R* = 0.012
		*p* = 0.260	*p* = 0.036	*p* = 0.881	*p* = 0.932
		*p*_fdr_ = 0.513	*p*_fdr_ = 0.361	*p*_fdr_ = 0.932	*p*_fdr_ = 0.932
Total cholesterol (mmol/L)	*R* = −0.161	1.000	*R* = −0.081	*R* = 0.066	*R* = 0.094
	*p* = 0.260		*p* = 0.570	*p* = 0.647	*p* = 0.513
	*p*_fdr_ = 0.513		*p*_fdr_ = 0.647	*p*_fdr_ = 0.647	*p*_fdr_ = 0.513
Creatinine (µmol/L)	*R* = −0.294	*R* = −0.081	1.000	*R* = 0.218	*R* = 0.003
	*p* = 0.036	*p* = 0.570		*p* = 0.124	*p* = 0.982
	*p*_fdr_ = 0.361	*p*_fdr_ = 0.647		*p*_fdr_ = 0.419	*p*_fdr_ = 0.982
Insulin (µIU/mL)	*R* = 0.022	*R* = 0.066	*R* = 0.218	1.000	*R* = 0.196
	*p* = 0.881	*p* = 0.647	*p* = 0.124		*p* = 0.168
	*p*_fdr_ = 0.932	*p*_fdr_ = 0.647	*p*_fdr_ = 0.419		*p*_fdr_ = 0.419
1,5 AG (ng/mL)	*R* = 0.012	*R* = 0.094	*R* = 0.003	*R* = 0.196	1.000
	*p* = 0.932	*p* = 0.513	*p* = 0.982	*p* = 0.168	
	*p*_fdr_ = 0.932	*p*_fdr_ = 0.513	*p*_fdr_ = 0.982	*p*_fdr_ = 0.419	

Note: 1,5-AG—1,5-anhydroglucitol, *R*—Spearman’s rank correlation coefficient, *p*_fdr_—*p*-value adjusted for multiple comparisons using the Benjamini–Hochberg false discovery rate procedure.

## Data Availability

The original contributions presented in this study are included in the article. Further inquiries can be directed to the corresponding author.
